# Supplementary material for: Cobalt Nanoparticles Embedded into N-Doped Carbon from Metal Organic Frameworks as Highly Active Electrocatalyst for Oxygen Evolution Reaction
Source: Polymers (Basel). 2019 May 8;11(5):828. doi: 10.3390/polym11050828 (PMC6572389; doi:10.3390/polym11050828)
Supplement: Supplementary file 1 [file polymers-11-00828-s001.pdf]

Supporting Information

Cobalt nanoparticles embedded into N-doped carbon from metal organic frameworks as highly active electrocatalyst for oxygen evolution reaction

Jitao Lu[a], Yue Zeng[a], Xiaoxue Ma[a], Huiqin Wang[a], Linna Gao[b], Qingguo Meng[a,c] *

---

1. Materials and methods

All chemicals were of reagent grade purity and purchased from commercial vendors without further purification. Powder X-ray diffraction (PXRD) measurements were carried out on a Bruker AXS D8 Advance instrument. The specific surface area were measured on a Bel Japan BELSORP-MINIII. Transmission electron microscopic (TEM) images were measured on Hitachi JSM-7500F. Scanning electron microscopic (SEM) images were obtained on a Hitachi JEM-2100F. X-ray photoelectron spectroscopy (XPS) was collected on Thermo Scientific ESCALAB 250Xi using Al Ka radiation and the C 1s peak at 284.8 eV as the internal standard. All electrochemical measurements were performed on a CHI760E electrochemical workstation.

2. Experiment for electrochemical measurements

A conventional three-electrode system was used with Cu Foam electrode as work electrode, Saturated calomel electrode (SCE) as the reference electrode and Platinum (Pt) foil as counter electrode (surface area of 15 x 15 mm). The potential values are corrected to the reverse hydrogen electrode (RHE) according the equation E (RHE) = E (SCE) + 0.245 + 0.0591 pH V. Typically, 5.0 mg of **1** and 20.0 μL Nafion solution (5 wt%) were dispersed in 1 ml of mixed solvents of deionized water and ethanol (V\V = 1:1) to form a homogeneous solution. Then 10.0 μL of the homogeneous solution was put on a Cu foam electrode to prepare the work electrode. In order to achieve a constant state of the anodic electrode, the catalyst was electrochemically pre-activated by 15 cyclic voltammetry scans at a scan rate of 10 mV s^{-1} before the electrochemical test.

3. The powder XRD pattern of Co-MOFs

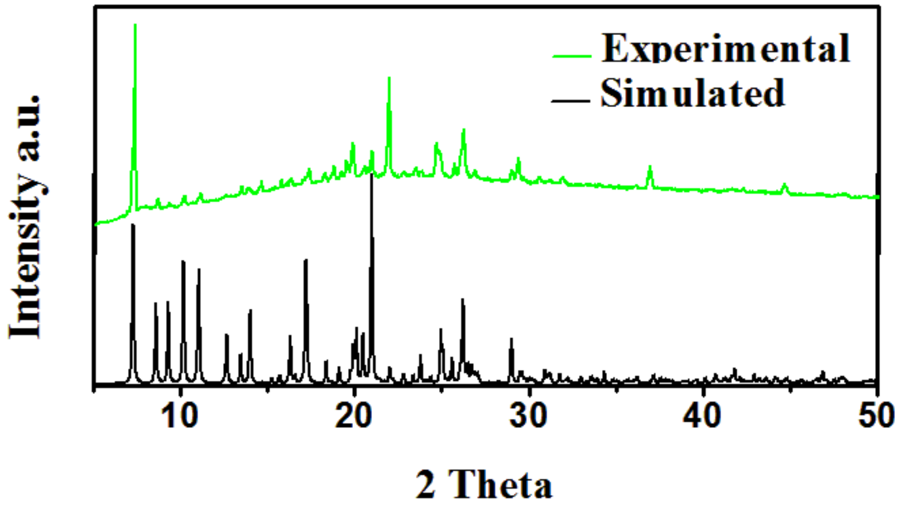

Figure S1. The powder XRD pattern and the simulated one from the single-crystal diffraction data of Co-MOFs.

4. The pore size distribution plot of Co-NPs@NC-600

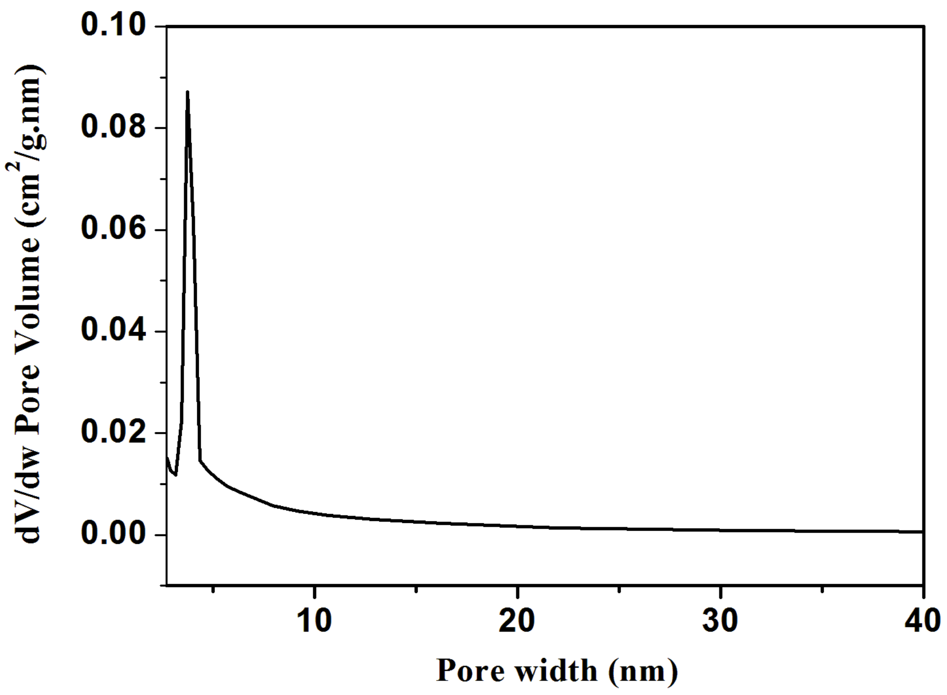

Figure S2. 4. The pore size distribution plot of Co-NPs@NC-600.

5. FTIR spectra of (A) Co-MOF and (B) Co-NPs@NC-600

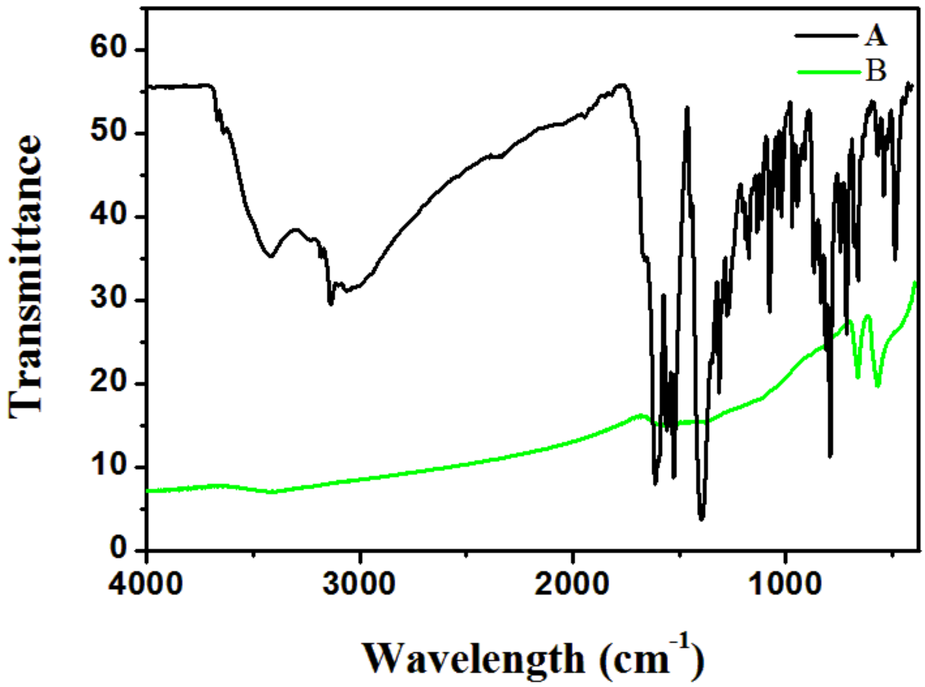

Figure S3. FTIR spectra of (A) Co-MOF and (B) Co-NPs@NC-600.

6. TGA curves of Co-NPs@NC-500, 600 and 700 samples measured in oxygen atmosphere

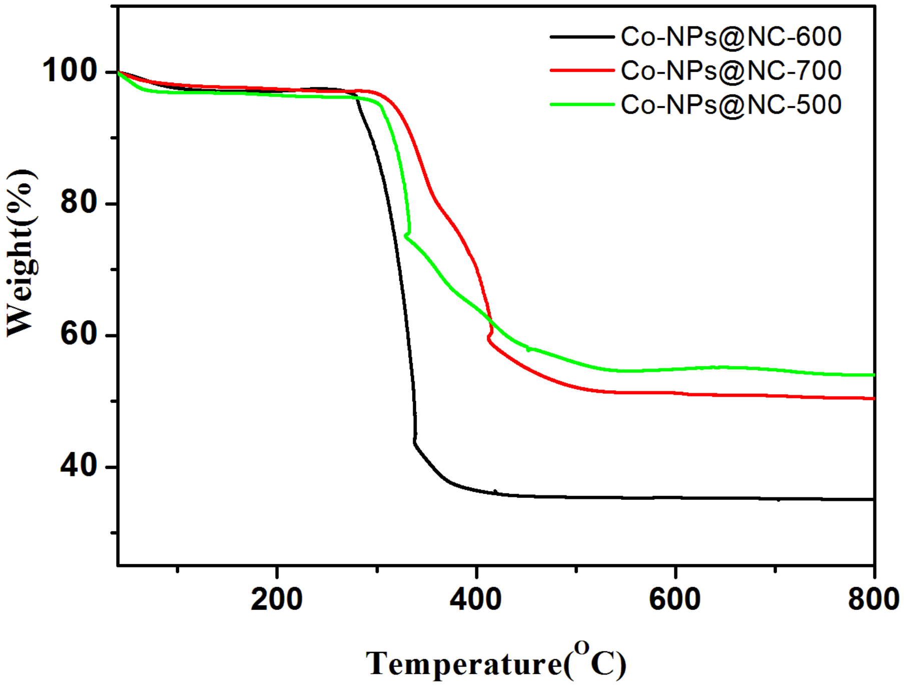

Figure S4. TGA curves of Co-NPs@NC-500, 600 and 700 samples measured in oxygen atmosphere.

7. SEM images of Co-NPs@NC-500 and 700 samples

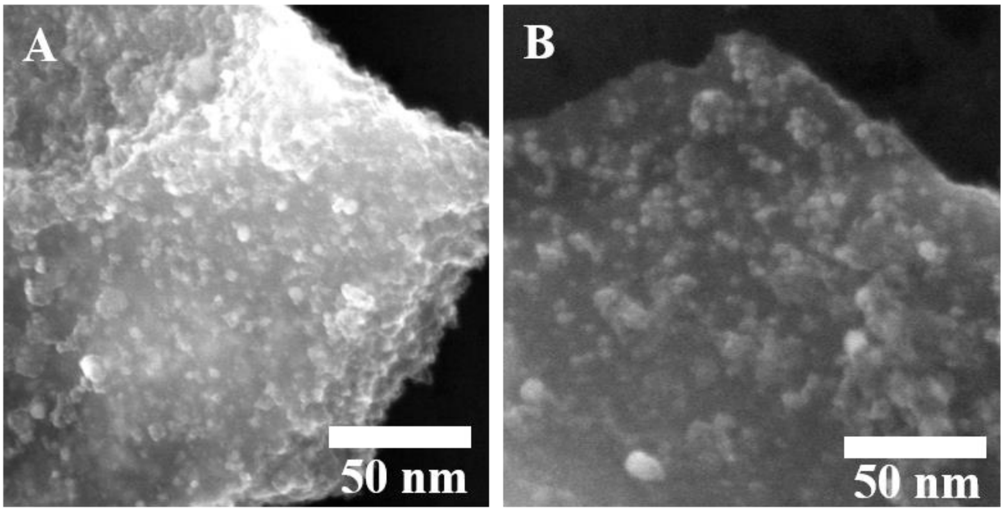

Figure S5. SEM images of Co-NPs@NC-500 (A) and 700 (B) samples.

8. The statistical analysis of Co nanoparticles.

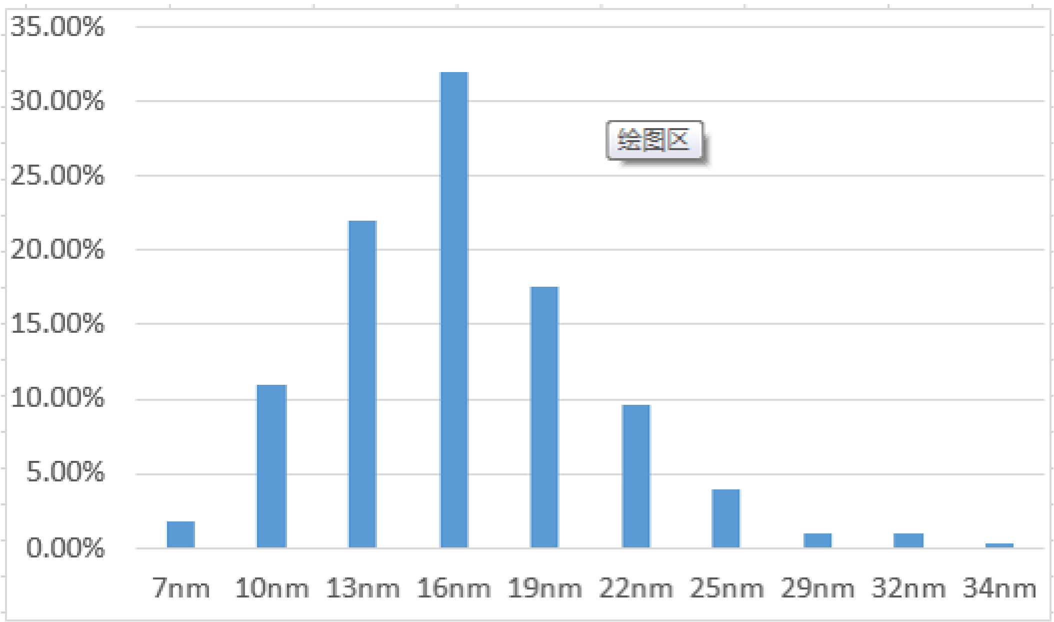

Fig. S6. The statistical analysis of Co nanoparticles.

9. The equivalent circuit of the EIS curves.

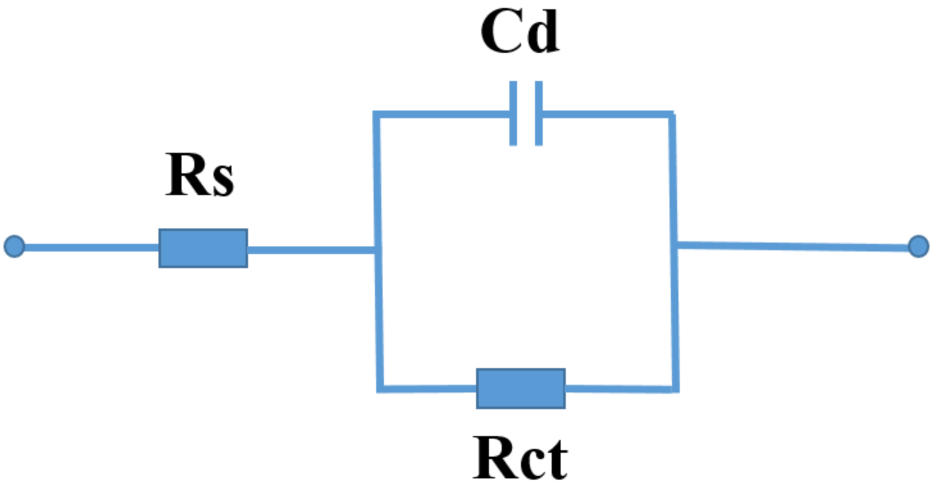

Figure S7. The equivalent circuit of the EIS curves.

10. Cyclic voltammograms at various scan rate of commercial Co powder.

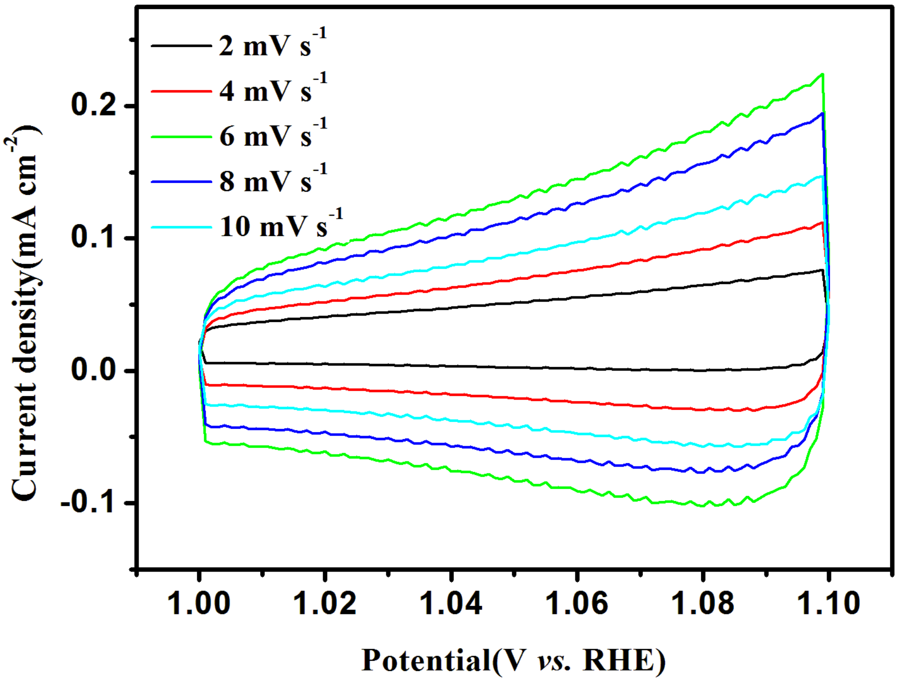

Fig. S8. Cyclic voltammograms at various scan rate of commercial Co powder.

11. Comparison of OER catalytic performance of recently reported state of the art Co-based OER catalysts

Table S1. Comparison of OER catalytic performance of recently reported state of the Co-based OER catalysts.

Catalysts	Overpotential @ 10mA cm^{-2} (mV)	Electrolyte	reference
Co@NC	292 365	1 M KOH 292	1
Co-Fe alloys	330	1 M KOH	2
Co-NG-5010-08	440	1 M KOH	3
NG-$CoSe_2$ nanobelt	370	1 M KOH	4
ultrathin $CoSe_2$	320	0.1M NaOH	5
Co_3O_4/NRGO	420	1 M KOH	6
hollow Co_3O_4 microtubes	290	1 M KOH	7
Co_3O_4 nanosheets	300	0.1 M KOH	8
Co_2P nanoneedles	310	1 M KOH	9
CoNPs-N-GR	400	1 M KOH	10
Co/CoP－HNC	300	1 M KOH	11
Co-NPs@NC	310	1 M KOH	Present work

1. Z. Wei, Y. Yang, M. Liu, J. Dong, X. Fan, X. Zhang. Cobalt nanocrystals embedded into N-doped carbon as highly active bifunctional electrocatalysts from pyrolysis of triazolebenzoate complex. Electrochim. Acta, 284, 2018, 733-741.

2. M. Xiong, D. Ivey.Composition effects of electrodeposited Co-Fe as electrocatalysts for the oxygen evolution reaction. Electrochim. Acta, 260, 2018, 872-881.

3. Y. Zhang, W.Li, L. Lu, W. Song, C. Wang, L. Zhou, J. Liu, Y. Chen, H. Jin, Y. Zhang, Tuning active sites on cobalt/nitrogen doped graphene for electrocatalytic hydrogen and oxygen evolution. Electrochim. Acta, 265, 2018, 497-506.

4. 7. M. Gao, X. Cao, Q. Gao, Y. Xu, Y. Zheng, J. Jiang, S.H. Yu, Nitrogen-Doped Graphene Supported $CoSe_2$ Nanobelt Composite Catalyst for Efficient Water Oxidation, ACS Nano. 8 (2014) 3970-3978.

5. 9. Y. Liu, H. Cheng, M. Lyu, S. Fan, Q. Liu, W. Zhang, Y. Zhi, C. Wang, C. Xiao, S. Wei, Low Overpotential in Vacancy-Rich Ultrathin $CoSe_2$ Nanosheets for Water Oxidation, J. Am. Chem. Soc. 136 (2014) 15670-15675.

6. K. Kumar, C. Canaff, J. Rousseau, S. Arrii-Clacens, T.W. Napporn, A. Habrioux, K.B. Kokoh, Effect of the Oxide-Carbon Heterointerface on the Activity of Co_3O_4/NRGO Nanocomposites toward ORR and OER, J. Phys. Chem. C 120 (2016) 7949-7958.

7. Y.P. Zhu, T.Y. Ma, M. Jaroniec, S.Z. Qiao, Self-Templating Synthesis of Hollow Co3O4 Microtube Arrays for Highly Efficient Water Electrolysis, Angew. Chem. Int. Ed. 56 (2017) 1324-1328.

8. L. Xu, Q. Jiang, Z. Xiao, X. Li, J. Huo, S. Wang, L. Dai, Plasma-Engraved Co3O4 Nanosheets with Oxygen Vacancies and High Surface Area for the Oxygen Evolution Reaction, Angew. Chem. Int. Ed. 55 (2016) 5277-5281.

9. A. Dutta, A.K. Samantara, S.K. Dutta, B. Jena, N. Pradhan, Surface-Oxidized

Dicobalt Phosphide Nanoneedles as a Nonprecious, Durable, and Efficient OER Catalyst, ACS Energy Lett. 1 (2016) 169-174.
10. Q. Zhao, Q. Zhang, M. Fu, Y. Liu, Y. Sun, H. Lu, X. Fan, Y. Zhang, H. Wang, Highly dispersed cobalt decorated uniform nitrogen doped graphene derived from polydopamine positioning metal-organic frameworks for highly efficient electrochemical water oxidation. Electrochimica Acta 289 (2018) 139-148.
11. Y. Hao, Y. Xu, W. Liu, X. Sun, Co/CoP embedded in a hairy nitrogen-doped carbon polyhedron as an advanced tri-functional electrocatalyst. Mater. Horiz. 5 (2018) 108-115.
